# PCSK-9 Inhibitors and Cardiovascular Outcomes: A Systematic Review With Meta-Analysis

**DOI:** 10.7759/cureus.46605

**Published:** 2023-10-06

**Authors:** Adi prasad Bodapati, Ayesha Hanif, Donatus K Okafor, Gitika Katyal, Gursharan Kaur, Hafsa Ashraf, Safeera Khan

**Affiliations:** 1 Internal Medicine, California Institute of Behavioral Neurosciences & Psychology, Fairfield, USA; 2 Surgery, California Institute of Behavioral Neurosciences & Psychology, Fairfield, USA

**Keywords:** mortality, cardiovascular events, alirocumab, evolocumab, pcsk9 inhibitors

## Abstract

Proprotein convertase subtilisin/kexin type 9 (PCSK9) inhibitors have been approved to treat dyslipidaemia. However, there is a lack of knowledge on the most efficient PCSK9 therapies that target PCSK9 for secondary prevention in subjects at high risk for cardiovascular (CV) events. Thus, this study aimed to assess the efficacy and safety of anti-PCSK9 antibodies in randomized controlled trials (RCTs). A comprehensive review of the available literature was done to identify RCTs that compared the use of PCSK9 inhibitors coupled with placebo or ezetimibe for the secondary prevention of CV events in patients on statin-background therapy. All-cause mortality was the major efficacy endpoint, while severe adverse events were the key safety outcome. A random effects model was used, and data were presented as risk ratio (RR) or risk difference with their corresponding 95% confidence intervals (CI). The heterogeneity of the publications was determined using Cochran’s Q test, and publication bias was visually examined using funnel plots. All the chosen studies’ quality was assessed using the Critical Appraisal Checklists for Studies created by the Joanna Briggs Institute (JBI). Forty-one studies (76,304 patients: 49,086 on evolocumab, and 27,218 on alirocumab) were included, and their years of publication spanned from 2010 to 2023. Overall, no significant differences were observed in CV and all-cause mortality between PCSK9 inhibitors and controls. However, alirocumab use was linked to a reduced risk of all-cause death compared to control, but not evolocumab. Each of the drugs, evolocumab and alirocumab, significantly reduced the risk of myocardial infarction (MI), coronary revascularization, and ischemic stroke. In comparison to the control therapy, the risk of major detrimental sequelae was significantly reduced by alirocumab therapy in the subgroup analysis of each PCSK9 inhibitor, whereas evolocumab treatment did not demonstrate significant differences (RR = 0.88; 95% CI = 0.72-1.04; evolocumab: RR = 0.99; 95% CI = 0.87-1.11). Both evolocumab and alirocumab are well-tolerated, safe medications that significantly lower low-density lipoprotein (LDL) levels.

## Introduction and background

Patients with established cardiovascular (CV) disorders continue to have greater mortality risks due to recurrent CV events. The most common cause of death globally is atherosclerotic cardiovascular disease (ASCVD) [1,2]. In 2016, 5.52 million individuals died of cerebrovascular disease, and ischemic heart disease caused mortality in 9.48 million, according to the Global Burden of Illness Study [1]. The primary and secondary prevention of CV disease can both be improved by lipid-lowering medications. Dyslipidemia, particularly high low-density lipoprotein cholesterol (LDL-C), is a significant risk factor for ASCVD [3,4].

Statins have long been considered the first-line treatment for reducing cholesterol and averting future CV problems [5,6]. According to the most recent US and European recommendations, proprotein convertase subtilisin/kexin type 9 (PCSK9) inhibitors combined with ezetimibe and statin drugs are implied in lowered CV risk in these individuals. Because PCSK9 promotes the breakdown of LDL receptors, LDL cannot be cleared from circulation. Thus, by modulating LDL receptor expression on the hepatocytes’ surface, modulators that inhibit PCSK9 may decrease LDL and, subsequently, significant CV events [7]. When used with statins, PCSK9 medications have been demonstrated to improve CV outcomes. According to ODYSSEY OUTCOMES research data, adding alirocumab to maximally tolerated statin therapy reduces the risk of CV events. When used with the maximum tolerable dose of statin treatment, evolocumab mitigated the risk of CV events in individuals with ASCVD [8].

Both PCSK9 inhibitors (evolocumab and alirocumab), having received FDA approval in 2015, have been approved for use in people with existing CV disease to reduce the risk of stroke, myocardial infarction, and coronary revascularization [5]. Alirocumab and evolocumab are often safe, according to a prior meta-analysis of 25 randomized, controlled studies. Evolocumab was shown to minimize the frequency of abnormal liver function, but alirocumab was found to increase the frequency of injection-site responses [5]. However, there is a dearth of information on PCSK9 inhibitors’ impacts on CV outcomes. We aimed to conduct an updated meta-analysis to demonstrate the effectiveness of approved PCSK9 inhibitors on CV outcomes. Comprehending the efficiency of PCSK-9 inhibitors in lowering CV events such as heart attacks, strokes, and deaths from CV causes was the primary expected outcome.

## Review

Methods

This meta-analysis adheres to the prescribed guidelines outlined by the Preferred Reporting Items for Systematic Review and Meta-Analyses (PRISMA) checklist [9] and the Cochrane Handbook guidelines [10].

Search Strategy

Seven databases (PubMed, Science Direct, The Cochrane Library, Scopus, Web of Science, Embase, and Google Scholar) were searched in-depth for studies published from 2010 to 2023. During the literature search, no restrictions were exercised on the country or language of publication. Editorial letters, conference records, and practice recommendations were all excluded.

This systematic review and meta-analysis comprises all randomized clinical studies comparing PCSK9 inhibitors with placebo or other active drugs. The following key terms were used to identify relevant studies: (“PCSK-9” OR “Evolocumab” OR “Alirocumab” OR “Cardiovascular disease” OR “Dyslipidemia” OR “Low-density lipoprotein” OR “PCSK9 inhibitors”), and only research articles were retrieved and reviewed. All possible combinations of keywords were utilized.

Study Selection

Titles and abstracts were checked for eligibility after removing duplicates. We independently evaluated each identified abstract’s full-text article.

Criteria for Considering Studies

Published studies (randomized clinical trials (RCTs)) reporting PCSK9 inhibitors usage (alirocumab and evolocumab) as a main or additional treatment for regulating cholesterol levels were required to meet the inclusion criteria. Since the manufacturer of bococizumab abandoned it in 2016, we did not include studies that compared it to a placebo. In addition to greater rates of injection site responses and immunogenicity therapy compared to other medications in this class, discontinuation was caused by an unanticipated attenuation of LDL cholesterol-lowering benefits over time. The studies include adult patients (age ≥ 18) with established atherosclerotic CV diseases, coronary heart disease (CHD), or disease risk equivalent. In addition, the CV outcomes of interest, such as myocardial infarction (MI), major adverse cardiovascular events (MACE), stroke, CV mortality, or coronary revascularization, are well-defined. The exclusion criteria were (1) studies not related to the topic and not providing enough data; (2) studies without results; (3) non-English studies; and (4) case reports, commentaries, guidelines, editorials, reviews, book chapters, and letters to the editor.

Reference lists of earlier systematic reviews and meta-analyses were also surveyed for pertinent papers. Grey literature and unpublished research might both be considered. In the event of several publications from the same trial, the article with the most relevant data was considered the primary publication.

Data Extraction and Outcomes of Interest

Two independent reviewers acquired data from certain investigations. A discussion was used to settle any disputes. Data were extracted using a typical Excel spreadsheet. Authors, study design, year of publication, patient characteristics, the proportion of subjects with coronary artery disease, diabetes, and hypertension at enrolment, and intervention details were collected for each included study.

Study Quality Assessment

Using the Critical Appraisal Checklists for Studies from the Joanna Briggs Institute (JBI) [11], the quality of each selected study was evaluated. For a “yes” score of 49% or below, the risk of bias in the study was deemed high. Studies scoring 50-69% were considered to have a moderate risk of bias, while studies scoring 70% or more had a low risk. All the studies included were evaluated for the risk of bias and then classified accordingly (i.e., studies with low risk and high risk of bias and studies with some concerns). Disagreements, if any, between the two independent reviewers were addressed by discussion and consensus.

Statistical Analysis

Stata software (version 17; StataCorp LLC, College Station, Texas) was used to carry out this meta-analysis. Continuous data were expressed using means, medians, and relevant standard deviations or ranges. Additionally, for descriptive purposes, categorical variables were shown as percentages and integers. There was a pooled meta-analysis. Based on the approach described by DerSimonian and Laird, the heterogeneity between studies was evaluated using Cochran’s Q test. Low heterogeneity was defined as an I-square value of less than 25%, moderate heterogeneity as one between 25% and 50%, and high heterogeneity as one of more than 50%. All the variables were analyzed using a random effects model. Using funnel plots, publication bias was visually investigated. A P-value <0.05 was considered statistically significant.

Results

Identification and Description of Studies

**Figure 1:**
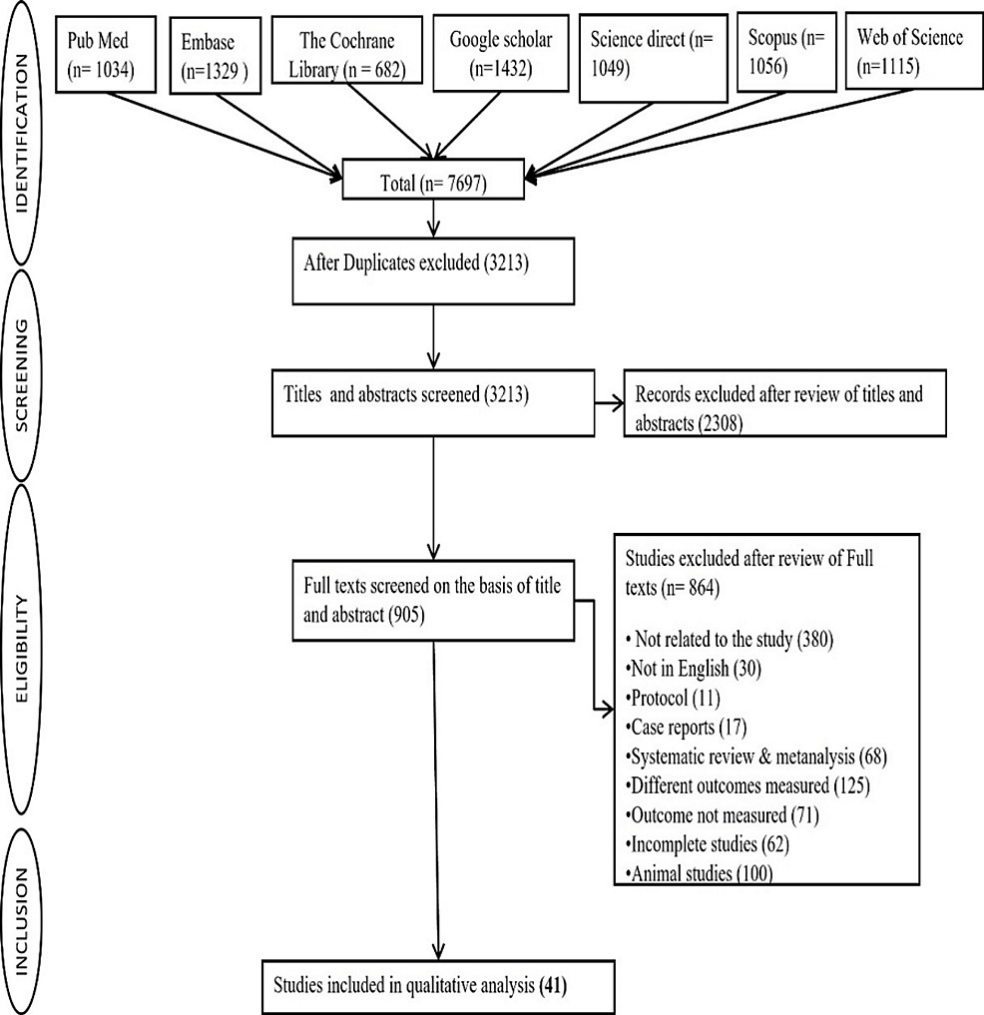
Flow chart depicting the selection of studies for analysis.

A total of 7,697 citations were identified, of which 3213 duplicate studies were eliminated. These included 1034 from PubMed, 1329 from Embase, 682 from The Cochrane Library, 1,432 from Google Scholar, 1,056 from Scopus, 1,049 from Science Direct, and 1,115 from Web of Science. After evaluating the titles and abstracts of 3,213 articles, a total of 2,308 studies were excluded. The remaining 905 articles met the requirements for the full-text review. Following the application of exclusion criteria, 864 complete texts were eliminated, leaving 41 articles for the final qualitative analysis. The flow diagram (Figure 1) depicts the study selection procedure.

Table 1 includes an overview of each research’s major demographic and clinical study characteristics [8,12-50]. The years of publication varied from 2012 to 2023, and the sample size was between 49 and 27,564. A sum total of 76,304 patients were enrolled in 41 RCTs. Evolocumab, a PCSK9 inhibitor, was used to treat 49,086 of them, and alirocumab was used for 27,218 patients. Table 2 presents a pooled estimate of baseline characteristics for all trials and each study drug.

**Table 1:** Basic characteristics of the included studies.

Study	Study design (RCT)	Sample size	Males (%)	Mean age (years)	Coronary artery disease (%)	Diabetes mellitus (%)	HTN (%)	Mean BMI (kg/m^2^)	Intervention	Treatment duration	Control type	Patients on statin (%)	Patients on ezetimibe (%)	Risk of bias
Schwartz et al. (2018) [8]	Phase III	18,924	74.8	58.6	100	28.8	64.7	NP	Alirocumab	208 weeks	Placebo	100	2.9	Low
Blom et al. (2014) [12]	Phase III	901	47.7	56.3	15.1	11.5	48.6	30.2	Evolocumab	52 weeks	Placebo	87.7	21	Moderate
Murphy et al. (2019) [13]	Phase III	27,564	75.4	62.5	100	36.6	80.1	NP	Evolocumab	113 weeks	Placebo	100	5.2	Low
Gaba et al. (2023) [14]	Phase III	6,559	76.8	61.9	83.9	34	83.4	30.1	Evolocumab	113 weeks	Placebo	76.8	5.8	Low
Sullivan et al. (2012) [15]	Phase II	157	36.3	61.8	17.2	13.4	47.1	28	Evolocumab	12 weeks	Placebo + Ezetimibe	15.9	39.5	Low
Stroes et al. (2018) [16]	Phase III	307	54.1	61.5	NP	20.2	59	NP	Evolocumab	12 weeks	Ezetimibe + placebo	17.9	33.2	Moderate
Stroes et al. (2018) [16]	Phase III	3,146	51.6	57.8	NP	NP	NP	NP	Evolocumab	12 weeks	Placebo/Ezetimibe	98.3	12.5	Low
Nissen et al. (2016) [17]	Phase III	218	51.4	58.8	31.7	11.9	51.4	28	Evolocumab	24 weeks	Ezetimibe	0	33.5	Moderate
Nicholls et al. (2016) [18]	Phase III	968	72.2	59.8	100	20.9	83	29.5	Evolocumab	76 weeks	Placebo	98.6	2.1	Low
Giugliano et al. (2012) [19]	Phase II	629	49.3	63.1	29.6	6.7	69.4	29	Evolocumab	12 weeks	Placebo	99.4	9	Low
Robinson et al. (2014) [20]	Phase III	1,896	54.2	60.1	22.5	15.5	NP	NP	Evolocumab	12 weeks	Ezetimibe, placebo	100	11.6	Moderate
McKenney et al. (2012) [21]	Phase II	182	47.5	56.7	5.5	12	44.8	29.2	Alirocumab	12 weeks	Placebo	100	NA	Low
Koren et al. (2012) [22]	Phase II	406	34	50.6	NA	0.2	31	30.8	Evolocumab	12 weeks	Placebo	0	11.1	Low
Koren et al. (2014) [23]	Phase III	614	31.1	52	NP	0.2	28.6	NP	Evolocumab	12 weeks	Placebo, Ezetimibe + placebo	0	12.5	Low
Moriarty et al. (2015) [24]	Phase III	313	54.8	63.4	46.5	23.9	62.7	29.2	Alirocumab	24 weeks	Ezetimibe	20.1	39.8	Low
Roth et al. (2016) [25]	Phase III	802	57.5	60.3	52.4	27	NP	30.9	Alirocumab	48 weeks	Placebo	68.1	24.7	Low
Stroes et al. (2016) [26]	Phase III	231	55.8	63.3	49.8	16.3	60.9	28.7	Alirocumab	24 weeks	Placebo	0	60.1	Low
Kereiakes et al. (2015) [27]	Phase III	314	65.8	63	78.2	43	NP	32.3	Alirocumab	52 weeks	Placebo	100	8.2	Low
Cannon et al. (2015) [28]	Phase III	720	73.6	61.6	90.1	30.7	NP	30.3	Alirocumab	52 weeks	Ezetimibe	99.9	33.5	Low
Ray et al. (2018) [29]	Phase IIIb	412	52.3	63.2	NA	100	88.1	32.9	Alirocumab	24 weeks	Standard Care	81.4	37.8	Moderate
Leiter et al. (2017) [30]	Phase IIIb	514	55.1	60.3	31.9	100	NP	31.2	Alirocumab	24 weeks	Placebo	74.9	15.1	Moderate
Moriarty et al. (2016) [31]	Phase III	62	58.1	58.7	79	16.1	NP	30.4	Alirocumab	18 weeks	Placebo	54.8	NA	Moderate
Kastelein et al. (2015) [32]	Phase III	485	55.1	52.6	42.7	9.1	39.6	28.8	Alirocumab	78 weeks	Placebo	100	57.2	Moderate
Ginsberg et al. (2016) [33]	Phase III	107	53	51	53	14.8	57.8	28.9	Alirocumab	78 weeks	Placebo	100	24.3	Low
Teramoto et al. (2016) [34]	Phase III	215	60.6	60.8	18.5	68.5	NP	25.5	Alirocumab	52 weeks	Placebo	100	NA	Low
Ako et al. (2019) [35]	Phase IV	206	70.9	61.2	14.6	28.2	61.7	25.1	Alirocumab	36 weeks	Standard Care	100	6.8	Low
Koh et al. (2018) [36]	Phase III	199	82.4	60.7	96.1	35.2	NP	26.5	Alirocumab	24 weeks	Placebo	100	13.1	Moderate
Robinson et al. (2015) [37]	Phase III	2,338	62.2	60	68.9	34.6	NP	30.4	Alirocumab	78 weeks	Placebo	100	14.3	Low
Roth et al. (2014) [38]	Phase III	103	53.4	45.2	NP	3.9	NP	29.3	Alirocumab	24 weeks	Ezetimibe	0	49.5	Moderate
Teramoto et al. (2019) [39]	Phase III	163	63.2	63.6	60	53.3	NP	25.9	Alirocumab	12 weeks	Placebo	34.3	19.6	Low
Bays et al. (2015) [40]	Phase III	354	65.1	62.8	56.3	49.9	78.3	31	Alirocumab	24 weeks	Ezetimibe or statin	100	28.7	Low
Farnier et al. (2016) [41]	Phase III	305	61.3	61	58	41.3	72.5	31.3	Alirocumab	24 weeks	Ezetimibe or statin	100	28.5	Low
Sabatine et al. (2015) [42]	Phase III	4,465	50.5	58	20.1	13.4	52	NP	Evolocumab	48 weeks	Standard therapy	70.1	13.5	Moderate
Roth et al. (2012) [43]	Phase II	92	40.2	56.9	3.3	14	51.1	29.5	Alirocumab	8 weeks	Placebo	100	NA	Low
Raal et al. (2012) [44]	Phase II	167	71.3	49.6	20.9	NP	NP	NP	Evolocumab	12 weeks	Placebo	89.8	64.7	Moderate
Raal et al. (2015) [45]	Phase III	329	58	50.6	31.1	NP	NP	NP	Evolocumab	12 weeks	Placebo	100	62	Low
Stein et al. (2012) [46]	Phase II	77	61	53.4	42	4	NP	29.1	Alirocumab	12 weeks	Placebo	100	71	Low
Teramoto et al. (2016) [47]	Phase II	100	45	57.7	1	16	35	24.7	Alirocumab	12 weeks	Placebo	100	NA	Moderate
Raal et al. (2015) [48]	Phase III	49	51	31	42.9	6.1	10.2	NP	Evolocumab	12 weeks	Placebo	100	91.8	Low
Hirayama et al. (2014) [49]	Phase II	307	62.9	61.5	25.1	38.1	73.6	NP	Evolocumab	12 weeks	Placebo	100	NA	Moderate
Kiyosue et al. (2016) [50]	Phase III	404	60.4	61.5	13	49	73.5	NP	Evolocumab	12 weeks	Placebo	100	NA	Moderate

**Table 2:** Pooled estimates of baseline characteristics across the included RCTs of PCSK9 inhibitors.

	Evolocumab studies (18)	Alirocumab studies (23)
Number of subjects	49,086	27,218
Age (years)	56.6 ± 7.8	58.9 ± 4.6
Males (%)	54.9	59.5
Coronary artery disease (%)	39.5	49.9
Hypertension (%)	56.5	59.8
Diabetes mellitus (%)	18.5	33.5
BMI (kg/m^2^)	29.4	29.1
Patients on statin (%)	69.7	79.7
Patients on ezetimibe (%)	26.8	29.7

Safety Outcomes

**Figure 2:**
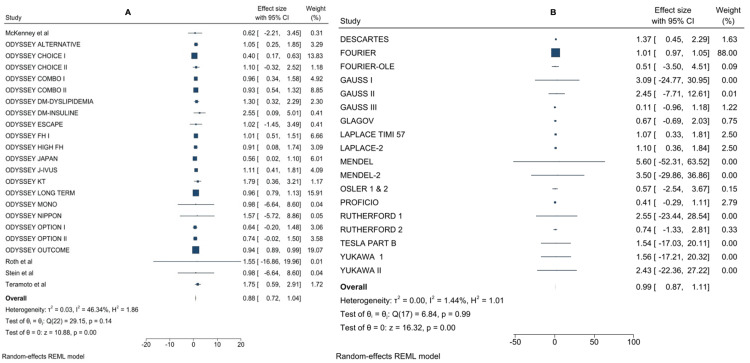
Forest plot comparing serious adverse events between PCSK9 inhibitors and control treatment: (A) evolocumab and (B) alirocumab.

To identify any adverse events related to the therapy, 41 studies were evaluated. There were no discernible changes between the two regimens. Alirocumab therapy significantly mitigated the risk of major adverse events in the subgroup analysis of each PCSK9 inhibitor when compared to the control treatment, whereas evolocumab treatment showed no such significant difference (alirocumab: RR = 0.88; 95% CI = 0.72-1.04; evolocumab: RR = 0.99; 95% CI = 0.87-1.11) (Figure 2). Compared to the control, alirocumab treatment was related to decreases in major adverse events. Neurocognitive problems and new-onset diabetes were not made more common by alirocumab and evolocumab treatments.

**Figure 3:**
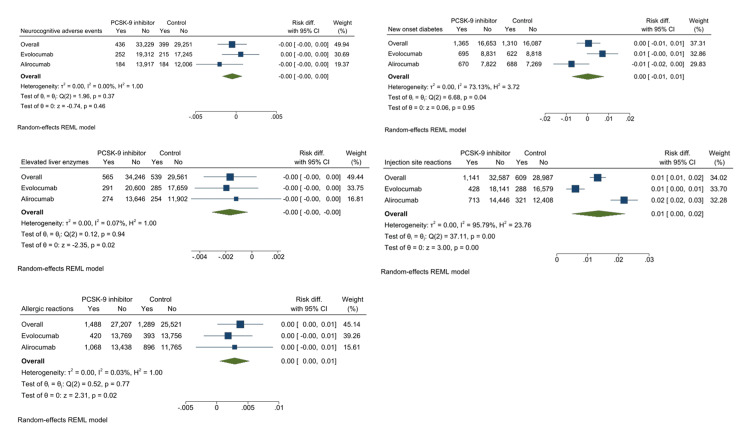
Forest plot comparing various serious adverse events between PCSK9 inhibitors and control treatment: (A) neurocognitive adverse events, (B) new-onset diabetes, (C) elevated liver enzymes, (D) injection site reactions, and (E) allergic reactions.

There were no significant differences in the risks of neurocognitive adverse events and new-onset diabetes; however, PCSK9 inhibitors were associated with higher allergy and injection site reactions than controls (Figure 3).

Efficacy Outcomes

**Figure 4:**
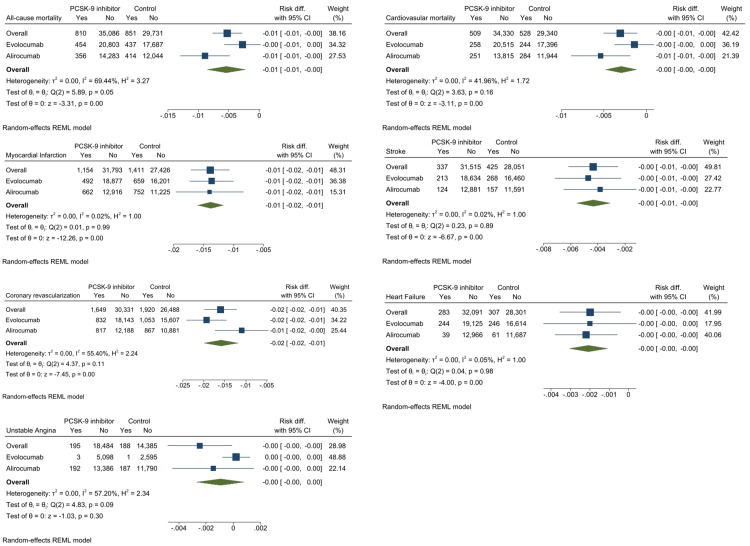
Efficacy endpoints for PCSK9 inhibitors vs. control. Results are reported as risk differences and 95% confidence intervals estimated using random-effect models.

Overall, no significant differences were discerned in CV and all-cause mortality between controls and PCSK9 inhibitors. However, using random effects models, alirocumab use was linked to a decreased risk of all-cause death when compared with control (Figure 4), but not the use of evolocumab. The use of PCSK9 inhibitors was linked to significantly lower rates of MI, ischemic stroke, and coronary revascularization when compared to controls. Evolocumab and alirocumab each had a personal relationship to a lower risk of coronary revascularization, MI, and ischemic stroke. The most effective treatment that reduces death from all causes is alirocumab. Alirocumab and evolocumab have been associated with lower MI rates. For lowering the risk of MI, evolocumab was rated as the most effective drug, while alirocumab was the most effective drug for reducing stroke risk.

Alirocumab was administered either monthly or biweekly, and both doses considerably decreased LDL levels. Biweekly 50-150 mg therapy reduced LDL levels by approximately 50% when compared to placebo, whereas monthly 150-300 mg treatment reduced LDL levels by a less noticeable amount when compared to ezetimibe. In the case of evolocumab, all six dosages at 12 weeks of follow-up significantly reduced LDL levels, with the highest reductions being attained with monthly doses of 420 mg and biweekly doses of 140 mg evolocumab compared to placebo.

Heterogeneity

The most common methods for detecting heterogeneity in meta-analysis include the Q test and the I2 index. An I2 score of 0% indicates that there is no between-study variability present in the analysis and that all variances are the product of sampling error. On the other hand, the closer an I2 index gets to 100%, the more the observed variance may be attributed to between-study variability, rather than just sampling error. Most of the outcomes in the studies included in this metanalysis showed significant heterogeneity.

Study Quality Assessment and Publication Bias

**Figure 5:**
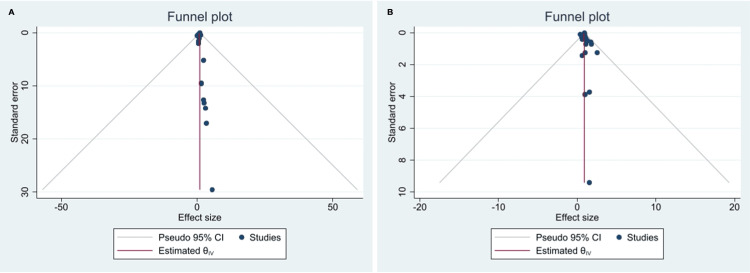
Funnel of studies comparing serious adverse events between PCSK9 inhibitors: (A) evolocumab and (B) alirocumab.

Two reviewers independently evaluated each included study’s quality. A moderate to low risk of bias is presented in most studies included in this analysis. The asymmetry in the funnel plots suggests that publication bias affects most of the results (Figure 5). Additionally, sensitivity analysis was carried out by recalculating all results without the information from each research included in the meta-analysis. The outcomes remained considerably unchanged throughout this process.

Discussion

CVD is substantially exacerbated by hypercholesterolemia. Statins are suggested as the first-line treatment for managing CVD since their introduction has significantly decreased CVD occurrences globally. However, there is still a need for other lipid-lowering medications, particularly those that lower LDL-C, as side effects of statins make it intolerable for some patients, making it difficult to achieve intensive LDL-C lowering due to extremely high baseline LDL-C levels, requiring more intensive lowering therapy due to their extremely high risk of CVD events [5,51,52]. Two PCSK9 inhibitors were compared for their comparative impact on CV outcomes in the current meta-analysis of 41 RCTs. The usage of alirocumab was linked to lower rates of major adverse events and all-cause mortality, according to the current research. Evolocumab treatment was also linked to a lower incidence of MI. Without raising serious safety issues, PCSK9 inhibitors were discovered to be the most successful medication for lowering CV events.

The drug of choice for treating hypercholesterolemia is statin therapy [53,54]. They lower LDL-C levels and, when administered for primary or secondary prevention, lower CV morbidity and death [3,55,56]. Patients with severe hypercholesterolemia or who respond inadequately to statin therapy are often advised to take other drugs, such as bile acid sequestrants, ezetimibe, and PCSK9 inhibitors, in addition to statin therapy [53,57]. PCSK9 inhibitors are completely human monoclonal antibodies that specifically target the PCSK9 protein and stop the PCSK9-LDL receptor from binding. These medications include evolocumab and alirocumab [52]. Evolocumab and alirocumab were both approved by the European Medicines Agency in July and September of 2015, respectively. Compared to statins, PCSK9 inhibitors result in a greater decrease in LDL cholesterol [51,58-61]. It is interesting to note that PCSK9 genetic variations linked to reduced LDL cholesterol are also linked to higher fasting glucose levels and an elevated risk of developing type 2 diabetes [62]. Understanding the precise effects of those medications on diabetic patients’ glucose and lipid metabolism is critical since diabetic people may make up a significant fraction of those obtaining a prescription for PCSK9 inhibitors.

Two pertinent clinical outcome trials, FOURIER (evolocumab) and, more recently, ODYSSEY OUTCOMES (alirocumab), have demonstrated the beneficial effects of PCSK9 inhibitors on CV outcomes when used in conjunction with statin therapy. The initial evidence of improved CV outcomes from evolocumab use originated from predefined exploratory data analysis from two extension trials (OSLER-1 and OSLER-2), comprising 4,465 patients, in total, who had successfully completed earlier phase 2 or 3 lipid-lowering studies [29,63]. In the OSLER investigations, patients were randomized to receive either conventional treatment alone or standard therapy in addition to evolocumab [59]. After a median follow-up of 11.1 months, the composite CV endpoint rate was significantly lower in the evolocumab group than in the control group (HR = 0.47, 95% CI = 0.28-0.78, p=0.003). This endpoint includes unstable angina, transient ischemic attack, MI, stroke, coronary revascularization, or heart failure and death. Evolocumab lowers the risk of CV events when combined with statin medication, according to conclusive findings from the FOURIER study [64].

Compared to a placebo at 48 weeks, evolocumab was linked to a 59% decrease in LDL-C values (p = 0.001). Evolocumab significantly decreased the risk of the main MACE endpoint by 15% compared to placebo after a median of 2.2 years (HR = 0.85, 95% CI = 0.79-0.92, p = 0.001). Additionally, it decreased the risk of several distinct outcomes from the primary goal, such as MI, stroke, and coronary revascularization, as well as the important secondary endpoint (a composite of MI, CV death, or stroke). There were no decreases in the risk of unstable angina, CV death, or overall mortality. The major endpoint’s risk was reduced from 12% in the first year to 19% thereafter, while the important secondary endpoint’s risk reduction went from 16% to 25% with time. According to the findings for the primary secondary endpoints, 74 people would need to be treated for a total of two years to avoid one of the primary secondary endpoint events (CV mortality, MI, or stroke) [64]. The frequencies of total adverse events, major adverse events, newly diagnosed diabetes, and allergic responses were not significantly different across the groups. Evolocumab injections resulted in higher injection-site responses than placebo injections (2.1% vs. 1.6%) [64].

Preliminary indications of the advantages of alirocumab in terms of CV outcomes were presented in the ODYSSEY LONG TERM study. A total of 2,341 individuals at high risk of CV events taking maximally tolerable statin medication and having an LDL-C level of less than 1.8 mmol/L (70 mg/dL) participated in this randomized, double-blind, phase 3 study [65]. Every two weeks for 78 weeks, patients were randomized to either a placebo or alirocumab 150 mg. The main objective, the percentage change in LDL-C at 24 weeks, was considerably higher in the alirocumab group. After 78 weeks, the alirocumab group had substantially fewer MACE (death from CHD, fatal or nonfatal ischemic stroke, nonfatal MI, or unstable angina requiring hospitalization) than the placebo group (1.7% versus 3.3%; HR = 0.52, 95% CI = 0.31-0.90, p = 0.02), according to a post hoc analysis. More conclusive findings came from the ODYSSEY OUTCOMES experiment [8].

Alirocumab was linked to substantial reductions in ischemic stroke, nonfatal MI, and unstable angina among the individual components of the main outcome but not in CHD mortality. In the alirocumab group, CV events, major CHD events, and a composite of death/nonfatal MI/nonfatal ischemic stroke were substantially less frequent, according to hierarchical testing of secondary objectives. Alirocumab was linked to a 15% relative decrease in all-cause mortality incidences (HR = 0.85, 95% CI = 0.73-0.98); however, due to the hierarchical testing strategy, this endpoint was not statistically evaluated [11]. There was little difference between the groups in the incidence of adverse events, including significant adverse events, newly diagnosed diabetes, or allergic responses. Alirocumab injections caused higher injection-site responses than placebo injections (3.8% vs. 2.1%) [8].

Before the release of the ODYSSEY OUTCOMES data, accessible meta-analyses on the impact of PCSK9 inhibitors on CV outcomes were carried out [6,51,60,66,67]. These investigations verified that PCSK9 inhibitors, as compared to no PCSK9 medication, lowered the risk of CV events, but they did not detect a meaningful effect on mortality. A Bayesian network meta-analysis of statins, ezetimibe, and PCSK9 inhibitors revealed that PCSK9 inhibitors had the best surface under the cumulative ranking curve (SUCRA) to prevent MACE (a combination of stroke, MI, and all-cause mortality; primary endpoint), followed by statins (SUCRA 75%) and ezetimibe plus statins (SUCRA 51%) [6]. Following statins, PCSK9 inhibitors were scored best for MI and stroke but second best for all-cause mortality and CV.

## Conclusions

PCSK9 inhibitors (evolocumab and alirocumab) decreased CV risk and were well-tolerated and safe. Therefore, we suggest that adding alirocumab and evolocumab to standard lipid-lowering therapy is effective and not linked to an increased likelihood of adverse events.

## References

[1] GBD 2016 Disease and Injury Incidence and Prevalence Collaborators (2017). Global, regional, and national incidence, prevalence, and years lived with disability for 328 diseases and injuries for 195 countries, 1990-2016: a systematic analysis for the Global Burden of Disease Study 2016. Lancet.

[2] Herrington W, Lacey B, Sherliker P, Armitage J, Lewington S (2016). Epidemiology of atherosclerosis and the potential to reduce the global burden of atherothrombotic disease. Circ Res.

[3] Cholesterol Treatment Trialists' (CTT) Collaboration (2010). Efficacy and safety of more intensive lowering of LDL cholesterol: a meta-analysis of data from 170,000 participants in 26 randomised trials. Lancet.

[4] Collins R, Reith C, Emberson J (2016). Interpretation of the evidence for the efficacy and safety of statin therapy. Lancet.

[5] Choi HD, Kim JH (2023). An updated meta-analysis for safety evaluation of alirocumab and evolocumab as PCSK9 inhibitors. Cardiovasc Ther.

[6] Khan SU, Talluri S, Riaz H (2018). A Bayesian network meta-analysis of PCSK9 inhibitors, statins and ezetimibe with or without statins for cardiovascular outcomes. Eur J Prev Cardiol.

[7] Wang X, Wen D, Chen Y, Ma L, You C (2022). PCSK9 inhibitors for secondary prevention in patients with cardiovascular diseases: a bayesian network meta-analysis. Cardiovasc Diabetol.

[8] Schwartz GG, Steg PG, Szarek M (2018). Alirocumab and cardiovascular outcomes after acute coronary syndrome. N Engl J Med.

[9] Knobloch K, Yoon U, Vogt PM (2011). Preferred reporting items for systematic reviews and meta-analyses (PRISMA) statement and publication bias. J Craniomaxillofac Surg.

[10] Higgins JPT, Green S (2019). Cochrane Handbook for Systematic Reviews of Interventions. 2nd Edition.

[11] (2023). JBI: Critical appraisal tools. https://jbi.global/critical-appraisal-tools.

[12] Blom DJ, Hala T, Bolognese M (2014). A 52-week placebo-controlled trial of evolocumab in hyperlipidemia. N Engl J Med.

[13] Murphy SA, Pedersen TR, Gaciong ZA (2019). Effect of the PCSK9 inhibitor evolocumab on total cardiovascular events in patients with cardiovascular disease: a prespecified analysis from the FOURIER trial. JAMA Cardiol.

[14] Gaba P, O'Donoghue ML, Park JG (2023). Association between achieved low-density lipoprotein cholesterol levels and long-term cardiovascular and safety outcomes: an analysis of FOURIER-OLE. Circulation.

[15] Sullivan D, Olsson AG, Scott R (2012). Effect of a monoclonal antibody to PCSK9 on low-density lipoprotein cholesterol levels in statin-intolerant patients: the GAUSS randomized trial. JAMA.

[16] Stroes E, Robinson JG, Raal FJ (2018). Consistent LDL-C response with evolocumab among patient subgroups in PROFICIO: a pooled analysis of 3146 patients from phase 3 studies. Clin Cardiol.

[17] Nissen SE, Stroes E, Dent-Acosta RE (2016). Efficacy and tolerability of evolocumab vs ezetimibe in patients with muscle-related statin intolerance: the GAUSS-3 randomized clinical trial. JAMA.

[18] Nicholls SJ, Puri R, Anderson T (2016). Effect of evolocumab on progression of coronary disease in statin-treated patients: the GLAGOV randomized clinical trial. JAMA.

[19] Giugliano RP, Desai NR, Kohli P (2012). Efficacy, safety, and tolerability of a monoclonal antibody to proprotein convertase subtilisin/kexin type 9 in combination with a statin in patients with hypercholesterolaemia (LAPLACE-TIMI 57): a randomised, placebo-controlled, dose-ranging, phase 2 study. Lancet.

[20] Robinson JG, Nedergaard BS, Rogers WJ (2014). Effect of evolocumab or ezetimibe added to moderate- or high-intensity statin therapy on LDL-C lowering in patients with hypercholesterolemia: the LAPLACE-2 randomized clinical trial. JAMA.

[21] McKenney JM, Koren MJ, Kereiakes DJ, Hanotin C, Ferrand AC, Stein EA (2012). Safety and efficacy of a monoclonal antibody to proprotein convertase subtilisin/kexin type 9 serine protease, SAR236553/REGN727, in patients with primary hypercholesterolemia receiving ongoing stable atorvastatin therapy. J Am Coll Cardiol.

[22] Koren MJ, Scott R, Kim JB (2012). Efficacy, safety, and tolerability of a monoclonal antibody to proprotein convertase subtilisin/kexin type 9 as monotherapy in patients with hypercholesterolaemia (MENDEL): a randomised, double-blind, placebo-controlled, phase 2 study. Lancet.

[23] Koren MJ, Lundqvist P, Bolognese M (2014). Anti-PCSK9 monotherapy for hypercholesterolemia: the MENDEL-2 randomized, controlled phase III clinical trial of evolocumab. J Am Coll Cardiol.

[24] Moriarty PM, Thompson PD, Cannon CP (2015). Efficacy and safety of alirocumab vs ezetimibe in statin-intolerant patients, with a statin rechallenge arm: the ODYSSEY ALTERNATIVE randomized trial. J Clin Lipidol.

[25] Roth EM, Moriarty PM, Bergeron J (2016). A phase III randomized trial evaluating alirocumab 300 mg every 4 weeks as monotherapy or add-on to statin: ODYSSEY CHOICE I. Atherosclerosis.

[26] Stroes E, Guyton JR, Lepor N (2016). Efficacy and safety of alirocumab 150 mg every 4 weeks in patients with hypercholesterolemia not on statin therapy: the ODYSSEY CHOICE II study. J Am Heart Assoc.

[27] Kereiakes DJ, Robinson JG, Cannon CP, Lorenzato C, Pordy R, Chaudhari U, Colhoun HM (2015). Efficacy and safety of the proprotein convertase subtilisin/kexin type 9 inhibitor alirocumab among high cardiovascular risk patients on maximally tolerated statin therapy: the ODYSSEY COMBO I study. Am Heart J.

[28] Cannon CP, Cariou B, Blom D (2015). Efficacy and safety of alirocumab in high cardiovascular risk patients with inadequately controlled hypercholesterolaemia on maximally tolerated doses of statins: the ODYSSEY COMBO II randomized controlled trial. Eur Heart J.

[29] Ray KK, Leiter LA, Müller-Wieland D (2018). Alirocumab vs usual lipid-lowering care as add-on to statin therapy in individuals with type 2 diabetes and mixed dyslipidaemia: the ODYSSEY DM-DYSLIPIDEMIA randomized trial. Diabetes Obes Metab.

[30] Leiter LA, Cariou B, Müller-Wieland D (2017). Efficacy and safety of alirocumab in insulin-treated individuals with type 1 or type 2 diabetes and high cardiovascular risk: the ODYSSEY DM-INSULIN randomized trial. Diabetes Obes Metab.

[31] Moriarty PM, Parhofer KG, Babirak SP (2016). Alirocumab in patients with heterozygous familial hypercholesterolaemia undergoing lipoprotein apheresis: the ODYSSEY ESCAPE trial. Eur Heart J.

[32] Kastelein JJ, Ginsberg HN, Langslet G (2015). ODYSSEY FH I and FH II: 78 week results with alirocumab treatment in 735 patients with heterozygous familial hypercholesterolaemia. Eur Heart J.

[33] Ginsberg HN, Rader DJ, Raal FJ (2016). Efficacy and safety of alirocumab in patients with heterozygous familial hypercholesterolemia and LDL-C of 160 mg/dl or higher. Cardiovasc Drugs Ther.

[34] Teramoto T, Kobayashi M, Tasaki H (2016). Efficacy and safety of alirocumab in japanese patients with heterozygous familial hypercholesterolemia or at high cardiovascular risk with hypercholesterolemia not adequately controlled with statins - ODYSSEY JAPAN randomized controlled trial. Circ J.

[35] Ako J, Hibi K, Kozuma K (2018). Effect of alirocumab on coronary atheroma volume in Japanese patients with acute coronary syndromes and hypercholesterolemia not adequately controlled with statins: ODYSSEY J-IVUS rationale and design. J Cardiol.

[36] Koh KK, Nam CW, Chao TH (2018). A randomized trial evaluating the efficacy and safety of alirocumab in South Korea and Taiwan (ODYSSEY KT). J Clin Lipidol.

[37] Robinson JG, Farnier M, Krempf M (2015). Efficacy and safety of alirocumab in reducing lipids and cardiovascular events. N Engl J Med.

[38] Roth EM, McKenney JM (2015). ODYSSEY MONO: effect of alirocumab 75 mg subcutaneously every 2 weeks as monotherapy versus ezetimibe over 24 weeks. Future Cardiol.

[39] Teramoto T, Kiyosue A, Ishigaki Y (2019). Efficacy and safety of alirocumab 150mg every 4 weeks in hypercholesterolemic patients on non-statin lipid-lowering therapy or lowest strength dose of statin: ODYSSEY NIPPON. J Cardiol.

[40] Bays H, Gaudet D, Weiss R (2015). Alirocumab as add-on to atorvastatin versus other lipid treatment strategies: ODYSSEY OPTIONS I randomized trial. J Clin Endocrinol Metab.

[41] Farnier M, Jones P, Severance R (2016). Efficacy and safety of adding alirocumab to rosuvastatin versus adding ezetimibe or doubling the rosuvastatin dose in high cardiovascular-risk patients: the ODYSSEY OPTIONS II randomized trial. Atherosclerosis.

[42] Sabatine MS, Giugliano RP, Wiviott SD (2015). Efficacy and safety of evolocumab in reducing lipids and cardiovascular events. N Engl J Med.

[43] Roth EM, McKenney JM, Hanotin C, Asset G, Stein EA (2012). Atorvastatin with or without an antibody to PCSK9 in primary hypercholesterolemia. N Engl J Med.

[44] Raal F, Scott R, Somaratne R, Bridges I, Li G, Wasserman SM, Stein EA (2012). Low-density lipoprotein cholesterol-lowering effects of AMG 145, a monoclonal antibody to proprotein convertase subtilisin/kexin type 9 serine protease in patients with heterozygous familial hypercholesterolemia: the Reduction of LDL-C with PCSK9 Inhibition in Heterozygous Familial Hypercholesterolemia Disorder (RUTHERFORD) randomized trial. Circulation.

[45] Raal FJ, Stein EA, Dufour R (2015). PCSK9 inhibition with evolocumab (AMG 145) in heterozygous familial hypercholesterolaemia (RUTHERFORD-2): a randomised, double-blind, placebo-controlled trial. Lancet.

[46] Stein EA, Gipe D, Bergeron J (2012). Effect of a monoclonal antibody to PCSK9, REGN727/SAR236553, to reduce low-density lipoprotein cholesterol in patients with heterozygous familial hypercholesterolaemia on stable statin dose with or without ezetimibe therapy: a phase 2 randomised controlled trial. Lancet.

[47] Teramoto T, Kobayashi M, Uno K (2016). Efficacy and safety of alirocumab in Japanese subjects (phase 1 and 2 studies). Am J Cardiol.

[48] Raal FJ, Honarpour N, Blom DJ (2015). Inhibition of PCSK9 with evolocumab in homozygous familial hypercholesterolaemia (TESLA Part B): a randomised, double-blind, placebo-controlled trial. Lancet.

[49] Hirayama A, Honarpour N, Yoshida M, Yamashita S, Huang F, Wasserman SM, Teramoto T (2014). Effects of evolocumab (AMG 145), a monoclonal antibody to PCSK9, in hypercholesterolemic, statin-treated Japanese patients at high cardiovascular risk--primary results from the phase 2 YUKAWA study. Circ J.

[50] Kiyosue A, Honarpour N, Kurtz C, Xue A, Wasserman SM, Hirayama A (2016). A phase 3 study of evolocumab (AMG 145) in statin-treated Japanese patients at high cardiovascular risk. Am J Cardiol.

[51] Bai J, Gong LL, Li QF, Wang ZH (2018). Long-term efficacy and safety of proprotein convertase subtilisin/kexin 9 monoclonal antibodies: a meta-analysis of 11 randomized controlled trials. J Clin Lipidol.

[52] Chaudhary R, Garg J, Shah N, Sumner A (2017). PCSK9 inhibitors: a new era of lipid lowering therapy. World J Cardiol.

[53] Catapano AL, Graham I, De Backer G (2016). 2016 ESC/EAS guidelines for the management of dyslipidaemias. Eur Heart J.

[54] Stone NJ, Robinson JG, Lichtenstein AH (2014). 2013 ACC/AHA guideline on the treatment of blood cholesterol to reduce atherosclerotic cardiovascular risk in adults: a report of the American College of Cardiology/American Heart Association Task Force on Practice Guidelines. Circulation.

[55] Last AR, Ference JD, Menzel ER (2017). Hyperlipidemia: drugs for cardiovascular risk reduction in adults. Am Fam Physician.

[56] Taylor F, Huffman MD, Macedo AF (2013). Statins for the primary prevention of cardiovascular disease. Cochrane Database Syst Rev.

[57] Lloyd-Jones DM, Morris PB, Ballantyne CM (2017). 2017 focused update of the 2016 ACC expert consensus decision pathway on the role of non-statin therapies for LDL-cholesterol lowering in the management of atherosclerotic cardiovascular disease risk: a report of the American College of Cardiology Task Force on expert consensus decision pathways. J Am Coll Cardiol.

[58] Lipinski MJ, Benedetto U, Escarcega RO (2016). The impact of proprotein convertase subtilisin-kexin type 9 serine protease inhibitors on lipid levels and outcomes in patients with primary hypercholesterolaemia: a network meta-analysis. Eur Heart J.

[59] Navarese EP, Frediani L, Kandzari DE (2021). Efficacy and safety of intracoronary epinephrine versus conventional treatments alone in STEMI patients with refractory coronary no-reflow during primary PCI: the RESTORE observational study. Catheter Cardiovasc Interv.

[60] Schmidt AF, Pearce LS, Wilkins JT, Overington JP, Hingorani AD, Casas JP (2017). PCSK9 monoclonal antibodies for the primary and secondary prevention of cardiovascular disease. Cochrane Database Syst Rev.

[61] Sattar N, Preiss D, Robinson JG (2016). Lipid-lowering efficacy of the PCSK9 inhibitor evolocumab (AMG 145) in patients with type 2 diabetes: a meta-analysis of individual patient data. Lancet Diabetes Endocrinol.

[62] Zewinger S, Kleber ME, Tragante V (2017). Relations between lipoprotein(a) concentrations, LPA genetic variants, and the risk of mortality in patients with established coronary heart disease: a molecular and genetic association study. Lancet Diabetes Endocrinol.

[63] Giugliano RP, Mach F, Zavitz K (2017). Cognitive function in a randomized trial of evolocumab. N Engl J Med.

[64] Sabatine MS, Giugliano RP, Keech AC (2017). Evolocumab and clinical outcomes in patients with cardiovascular disease. N Engl J Med.

[65] Robinson JG, Farnier M, Krempf M (2015). Efficacy and safety of alirocumab in reducing lipids and cardiovascular events. N Engl J Med.

[66] Karatasakis A, Danek BA, Karacsonyi J (2017). Effect of PCSK9 inhibitors on clinical outcomes in patients with hypercholesterolemia: a meta-analysis of 35 randomized controlled trials. J Am Heart Assoc.

[67] Bajaj NS, Patel N, Kalra R, Ahmad A, Venkatraman A, Arora G, Arora P (2018). Neurological effects of proprotein convertase subtilisin/kexin type 9 inhibitors: direct comparisons. Eur Heart J Qual Care Clin Outcomes.

